# Granulosa Cell Apoptosis in the Ovarian Follicle—A Changing View

**DOI:** 10.3389/fendo.2018.00061

**Published:** 2018-03-02

**Authors:** Sheena L. P. Regan, Phil G. Knight, John L. Yovich, Yee Leung, Frank Arfuso, Arun Dharmarajan

**Affiliations:** ^1^Stem Cell and Cancer Biology Laboratory, School of Pharmacy and Biomedical Sciences, Curtin Health Innovation Research Institute, Curtin University, Perth, WA, Australia; ^2^School of Biological Sciences, University of Reading, Reading, United Kingdom; ^3^PIVET Medical Centre, Perth, WA, Australia; ^4^Western Australian Gynaecologic Cancer Service, King Edward Memorial Hospital for Women, Perth, WA, Australia

**Keywords:** apoptosis signaling, ovarian reserve, aging effects, fertility preservation, receptor of follicle stimulating hormone, bone morphogenetic proteins, mitogenic growth

## Abstract

Recent studies challenge the previous view that apoptosis within the granulosa cells of the maturing ovarian follicle is a reflection of aging and consequently a marker for poor quality of the contained oocyte. On the contrary, apoptosis within the granulosa cells is an integral part of normal development and has limited predictive capability regarding oocyte quality or the ensuing pregnancy rate in *in vitro* fertilization programs. This review article covers our revised understanding of the process of apoptosis within the ovarian follicle, its three phenotypes, the major signaling pathways underlying apoptosis as well as the associated mitochondrial pathways.

## Introduction

Pregnancy rate and oocyte quality have been linked to the incidence of apoptosis in women receiving *in vitro* fertilization (IVF) treatment (1). Poor prognosis patients (such as older women) had a greater incidence of apoptosis (number of pyknotic bodies), while follicles yielding oocytes that fertilized had lower levels of granulosa cell apoptosis (1, 2). Several growth factors and hormones are antiapoptotic, such as bone morphogenetic proteins (BMPs), follicle stimulating hormone (FSH), luteinizing hormone (LH), and estrogen. Recent studies from our group reported that granulosa cell expression of the receptors (R) of FSH (FSHR), BMP (BMPR1B), and LH (LHR) are reduced and dysregulated in older women; yet in the same cohort of women, granulosa cell apoptosis was highest in the younger rather than the older women (3–5). Different techniques have been applied historically to determine the level of apoptosis. In the light of recent reports for apoptosis analysis, there arises the question of whether apoptosis is an accurate measure for the interpretation of oocyte quality.

## Necrosis and Apoptosis: Morphological Features

**Figure 1:**
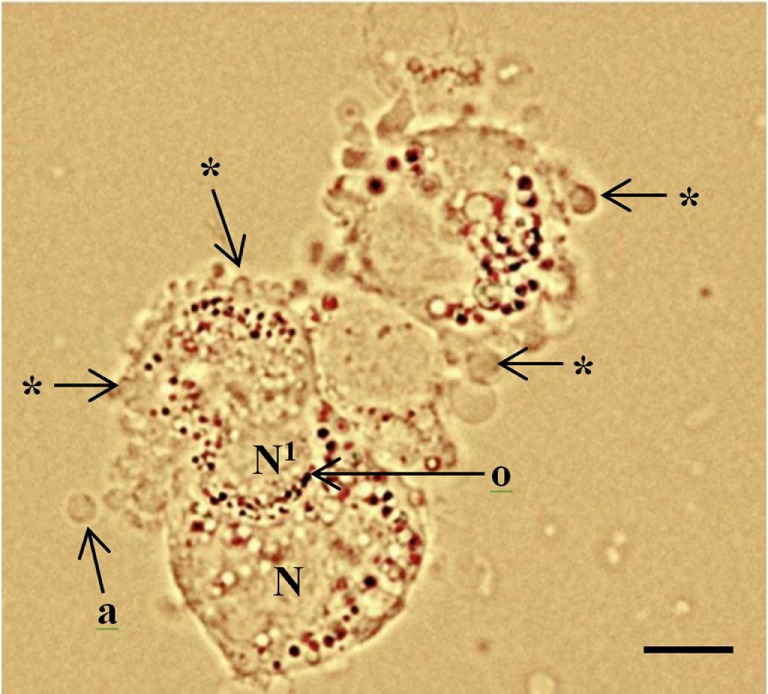
Periluteal granulosa cells and cell morphology. Human granulosa cells were collected from a 15 mm follicle during an *in vitro* fertilization cycle at the time of oocyte collection. The granulosa cells are peri-luteal cells; however, the cytoplasm is still relatively compact compared to granulosa cells collected from ovulatory follicles (5). The granulosa cells have dense clustering of organelles around the large round nucleus (N). The cytoplasm appears granular during the late stages of follicular phase; large lipid droplets contain hormones. Cytoplasmic extrusions or blebbing, which indicates late apoptosis are shown (*); apoptotic bodies (a); organelles (o) clustered around the nucleus. Healthy granulosa cell (N) without blebbing is engulfing a neighboring apoptotic granulosa cell nucleus (N1) *via* phagocytosis (10). Bar 5 µm.

Necrosis results from cellular exposure to a toxin or destructive agent which causes swelling and disruption to the cell’s organelles, leading to an irreversible breakdown of the cell’s membranes and the scattering of the cytoplasmic and nuclear contents (6). This leads to a marked inflammatory response by the body. However, apoptosis is caused by several distinctive signaling pathways, which culminate in shrinkage of the cell, cytoplasmic blebbing, and compartmentalization of organelles (Figure 1) (7–9). Characteristically there is no consequent inflammatory response. Although the cell membranes lose integrity, they fold and encapsulate (blebbing) to prevent the contents from affecting neighboring cells, a feature which is not observed in necrotic granulosa cells (10). Apoptotic cells typically have pyknotic, crescent-shaped or rounded dark bodies of dense DNA, fragmented into smaller sections. However, the majority of pyknotic cells in the middle layers of the membrana granulosa had been consumed by adjacent healthy cells.

**Figure 2:**
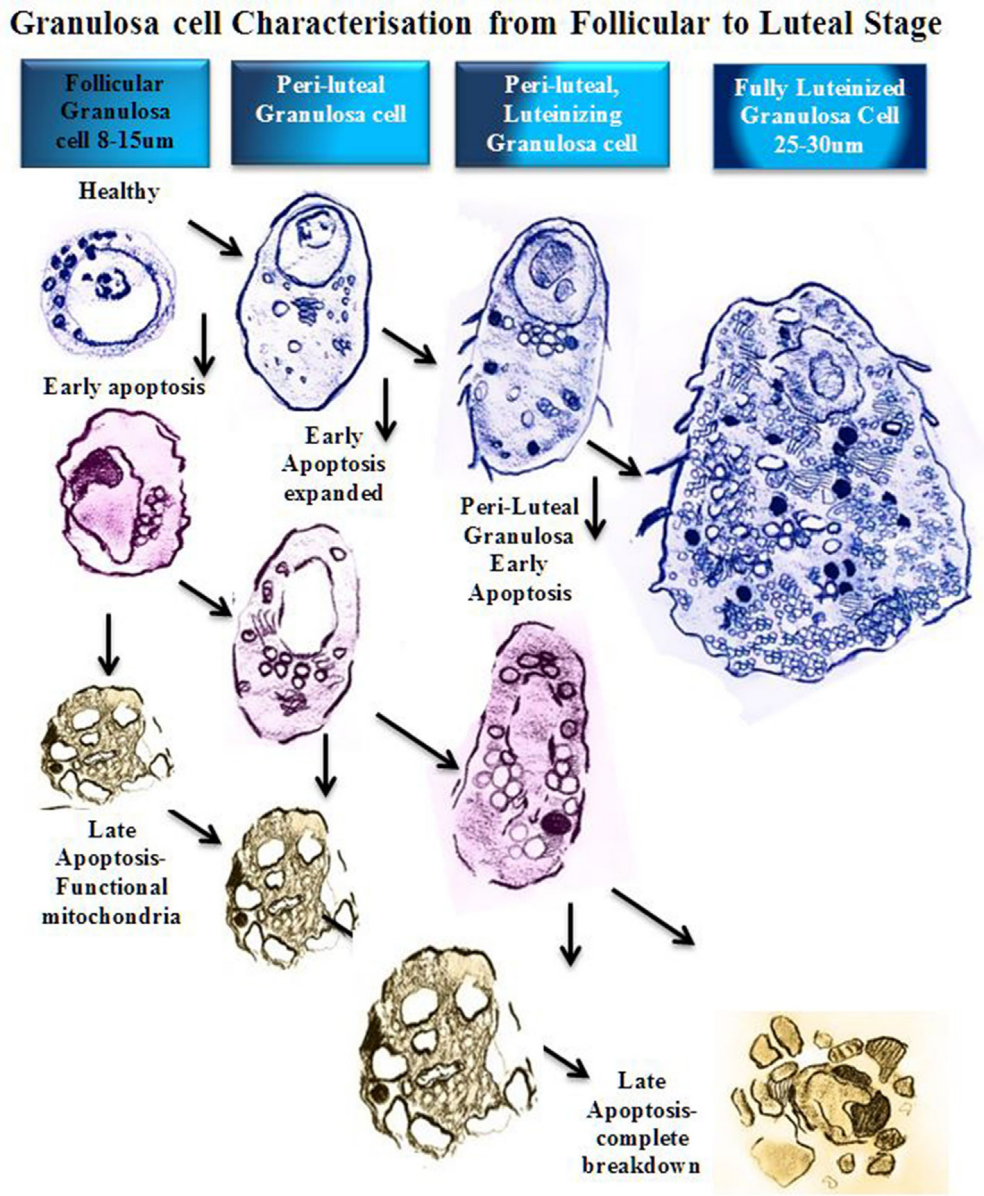
Schematic diagram of granulosa cell characterization from follicular to luteal phase. In a stage-dependent progression, the granulosa differentiates from a compact 8–15 µm cell with a large round nucleus and relatively small cytoplasm (12). The cytoplasm contains mitochondria, rough endoplasmic reticulum, Golgi apparatus, lipid droplets, and many other organelles (14). As the granulosa cell matures, the organelles proliferate and the cytoplasm expands to accommodate new steroidogenic capacity. A fully luteinized granulosa cell, 25–30 µm, contains a large volume of mitochondria, steroid filled lipid droplets, and smooth/rough endoplasmic reticulum, and has the capacity to produce progesterone directly (8). At any stage of follicular growth the granulosa cell can undergo apoptosis. Early apoptosis is characterized by collapse of the cell membranes and condensation of the chromatin, which often polarizes in the nucleus with the organelles clustered adjacent (15). Early apoptosis in a granulosa cell with an expanded cytoplasm is similarly changed with a greater volume of organelles clustered around a collapsing nucleus. Late stages of apoptosis end with compartmentalization of organelles into blebs and extrusion of apoptotic bodies that may contain nucleic matter.

Necrosis and apoptosis both culminate in cell death of the granulosa cell, and nucleic dyes that stain DNA material are commonly used to indicate the vitality of the cell membranes (11). Uniquely, the apoptotic granulosa cell will continue to synthesize steroid hormones until the mitochondrial membranes are disrupted. Functioning apoptotic granulosa cells undergo reorganization of the cell cytoplasm, creating blebs of non-cytoplasmic organelles at the periphery; and mitochondria, Golgi apparatus, and endoplasmic reticulum, which are clustered around the nucleus; large fluid filled vacuoles containing steroids, lipids, and proteins also accumulate (12, 13). The granulosa cell expands and reorganizes the contents of the cytoplasm, forming new organelles, particularly smooth endoplasmic reticulum (SER) for progesterone production (13). The SER and mitochondria are assembled in close association with the nucleus (8, 13). The mitochondria and large, round, lipid droplets are closely associated with the SER, all of which have substantially increased in number, and cluster around the nucleus to increase efficiency of steroid synthesis (Figure 2) (8).

During luteinization, granulosa cells form irregular microvilli and tight junctions between the cells, whereas in an apoptotic granulosa cell, the cell membrane disintegrates and spaces form (12). The apoptotic granulosa cell continues to produce steroids in large antral follicles until complete mitochondrial breakdown occurs (16).

## Granulosa Cell Apoptosis: Three Phenotypes, Their Initiation and Regulation

Atresia of ovarian follicles can be divided into three phenotypes, each with different mechanisms of initiation and regulation, but all involving granulosa cell apoptosis (17). The first, so-called “antral atresia”, affects the middle prolific layers of granulosa cells, with apoptosis progressing toward the antrum. The second, “basal atresia”, occurs in the granulosa cells closest to the basal lamina of very small antral follicles. The cells prematurely luteinize and begin to produce progesterone; however, they do not complete luteinization, with subsequent cell death (17). A third form of apoptosis in the preovulatory follicles is referred to as “terminal differentiation apoptosis” and is similar to epidermal skin cells sloughing off (10, 18). The granulosa cells that are sloughed off from the antral surface form globules that aggregate and float into the antral fluid (10). The globules average 40 µm in diameter and stain positively for propidium iodide (PI), which indicates that their cell membranes are compromised. However, the DNA is not fragmented, being of high molecular weight, and has a negative DNA fragmentation laddering result for the TUNEL (terminal deoxy-UPT nick end-labeling) assay. The majority of cell death in the membrana granulosa is *via* “antral atresia.” Apoptotic bodies and cytoplasmic blebbing are typical of antral apoptosis, whereas apoptotic cells in the middle or basement section of the granulosa membrana are commonly engulfed by neighboring healthy granulosa cells and/or infiltrating macrophages. There are three main areas of apoptosis inducement; growth factors, death receptors and cell damage. Apoptosis can occur at any stage of the development of the follicle. In small follicles the granulosa cells are compact with large round nuclei. As the follicle matures the predominantly estrogen producing granulosa differentiates steroidogenically, increasing the volume of mitochondria and SER. Apoptosis at this stage of development would similarly result in condensation of the nucleic contents and clustering of the organelles around the nucleus. In the late stages of apoptosis, cell membranes are broken down and the contents compartmentalize into apoptotic bodies.

## Major Apoptosis Signaling Pathways in the Ovarian Follicle

**Figure 3:**
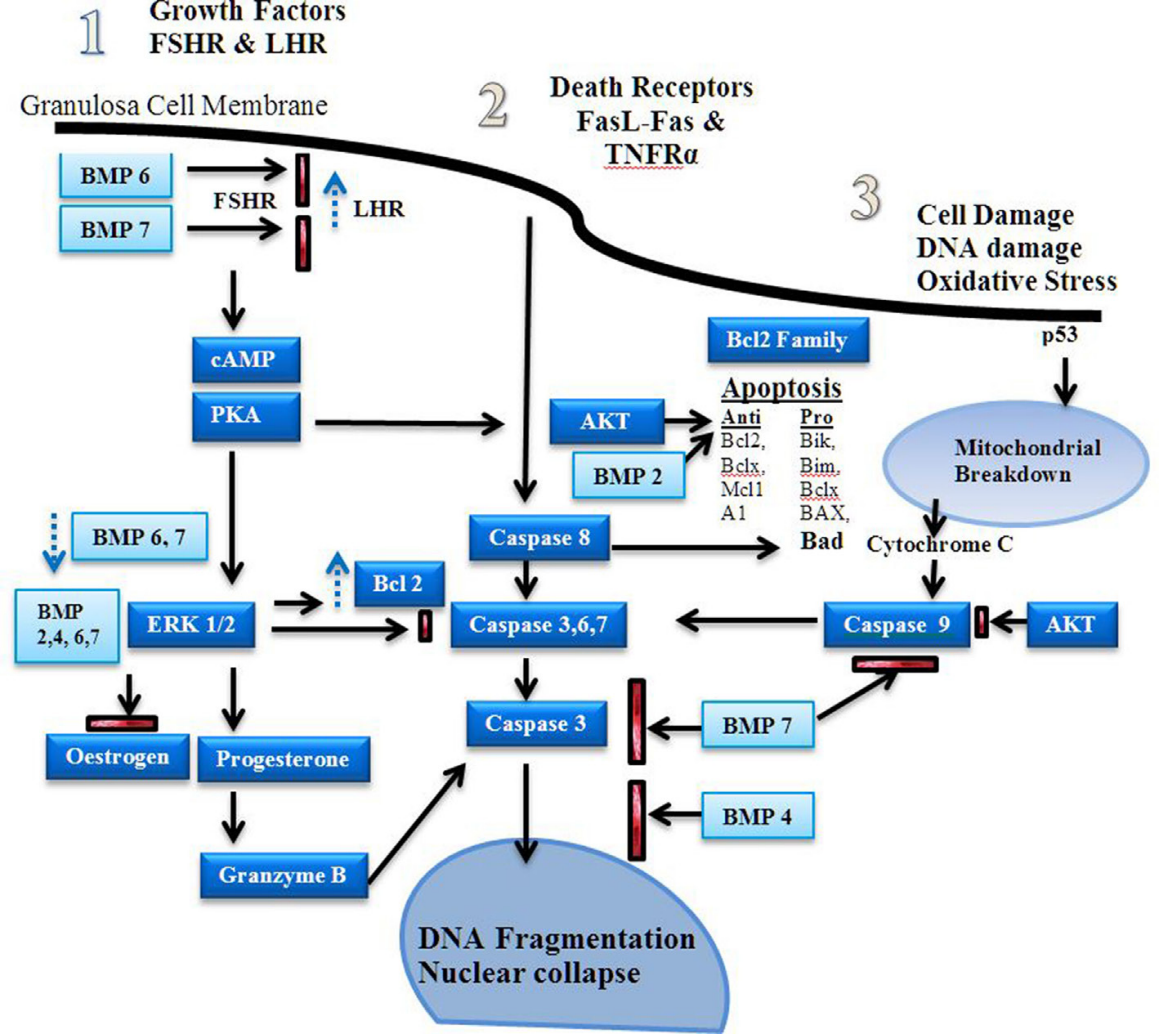
Apoptosis signaling pathways. Estrogen is a major driver of follicular growth and its effects results in inhibition of apoptosis. There are three main areas of apoptosis inducement: 1. growth factors, 2. death receptors, and 3. cell damage (20). Before antral cavity formation ovarian follicle androgens increase the receptor of follicle stimulating hormone (FSH) expression and high levels of BMPR1B activity, possibly *via* BMP6 and 7, suppress LHR expression (21). Antiapoptotic FSH induced cAMP–PKA promote ERK1/2 signaling, which increases Bcl-2 and promotes estrogen production in favor of progesterone synthesis (22–24). The bone morphogenetic proteins (BMPs) 2, 4, 6, and 7 inhibit progesterone synthesis, which reduces caspase 3, 6, and 7 production during the follicular phase (25). Dysregulation of the BMPs induces granzyme B synthesis leading to increased DNA fragmentation (16). Fas ligand (FasL) and TNFR activity induces caspase 8 induced DNA fragmentation (26). BMP 7 inhibits caspase 3 activity. Estrogen *via* the estrogen receptor has also been shown to reduce FasL activity (27, 28). Stress induced apoptosis *via* p53 causes mitochondrial breakdown *via* caspase 9 (29). Mitochondrial apoptosis is dependent on the ratio of pro and antiapoptotic factors of the Bcl-2 family (30). The Akt signaling pathway promotes antiapoptotic activity and is influenced by estrogen and growth factor-induced cAMP-PKA activity (31, 32).

There are three major signaling pathways for the development of apoptosis in granulosa cells (19). The first is growth factor-induced high levels of cAMP through granzyme B; the second affects mitochondrial function *via* the Bcl2 family member activation; and the third utilizes tumor necrosis factor alpha (TNFα) and Fas ligand (FasL)-Fas and other death receptors; all of which result in caspase-induced DNA fragmentation (Figure 3). In the ovary, granzyme B is stimulated by gonadotrophins and forskolin to bypass the mitochondria to preserve their function in the early stages of apoptosis, even though DNA fragmentation and nuclear collapse may have occurred (16).

Apoptosis can also be initiated by a number of extrinsic factors that may damage the cell such as DNA damage and oxidative stress, which activate p53-specific signaling pathways to trigger apoptotic mechanisms (8, 19, 33).

Conversely, many growth factors [such as insulin-like growth factor (IGF), epidermal growth factor (EGF), and fibroblast growth factor (FGF)] as well as gonadotrophins (FSH and LH) are antiapoptotic, which creates a microenvironment that ensures survival.

Estrogen and progesterone are the main antiapoptotic factors along with FSH, LH, EGF, IGF, FGF, prolactin, laminin, leptin, and glucocorticoids. The steroid producing capacity of a follicle is reflected in the hormone levels within serum and follicular fluid from the antral cavity of the follicle under the influence of steroidogenic acute regulatory protein (StAR) (34). The steroids produced by the theca and granulosa cells provide an antiapoptotic effect in a stage-specific manner during folliculogenesis.

After dominant follicle selection, the theca cell mitochondria convert cholesterol to pregnenolone by cytochrome P450 side-chain cleavage enzyme, mediated by StAR. The pregnenolone is transported out of the mitochondria and converted to progesterone by 3β-hydroxysteroid dehydrogen. The progesterone is then converted to androgens, predominantly androstenedione, dehydroepiandrosterone, and testosterone, and transported from the theca cell to the granulosa cell where it used to synthesize estrogen.

During the estrogen-driven proliferative phase of folliculogenesis, granulosa cell apoptosis is inhibited by antiapoptotic signaling pathways such as protein kinase B (Akt/PKB), extracellular signal-regulated kinase (ERK), EGF, and the BMPs. The Akt signaling pathway promotes antiapoptotic activity and is influenced by estrogen and growth factor induced cAMP-PKA activity. Estrogen directly inhibits FasL-induced apoptosis *via* the estrogen receptor, which activates the Akt–dependent pathway (20). In addition, the BMP ligands have been shown to inhibit apoptosis signaling down-stream of these signaling pathways in a number of studies (25, 35–38).

The LH surge-induced differentiation of the granulosa cell enables it to convert cholesterol to progesterone directly, and the levels rise substantially. At the time of ovulation the LH surge induces the granulosa cells to express progesterone receptors. The follicle undergoes progesterone-driven luteinization and enters the luteal phase, cell proliferation ceases, and high levels of progesterone inhibit apoptosis during corpus luteum formation (39).

## Mitochondrial Pathways of Apoptosis and the BMPs

Apoptosis is induced *via* a change in the balance of proapoptotic factors versus antiapoptotic factors. In most cells of the body, mitochondrial apoptosis is the primary pathway involved in programmed cell death. However, in the ovary, early apoptosis begins in the nucleus of granulosa cells, bypassing the mitochondria until the later stage of apoptosis. The B-cell lymphoma 2 gene (Bcl-2) family consists of proapoptotic and antiapoptotic factors (~40 known members) associated with the regulation of apoptosis *via* the mitochondria (27, 40).

Bone morphogenetic proteins have an inter-relationship with the proapoptotic factor Bax (a Bcl-2 derived prosurvival protein), and its ratio to antiapoptotic factors (Figure 3). Bcl-2 opposes Bax and its destruction of the mitochondrial membranes. Bax is present from the resting primordial follicle onward; whereas Bcl-2 is only present after primary to secondary transition, which may influence primordial follicle loss (41). BMP2, when overexpressed in renal tumors, signals *via* Smad 1, 5, and 8 (Smads, initially identified in drosophila, are signal transducers for receptors of the TGF-B superfamily), and increases GATA-4 expression, which inhibits Bax activity in the mitochondria (42). (The GATA transcription factor family are key mediators of sp hemopoietic progenitors.) In the mouse ovary, GATA-4 inhibits Bax-induced mitochondrial membrane permeabilization (41). Conversely, BMP2 induces apoptosis in bone by increasing caspase 3, 6, 7, and 9 expression (35), indicating a tissue-specific involvement of BMP2.

In the ovary BMP2 increases granulosal FSHRs in small follicles, and FSHR-mediated signaling is antiapoptotic in nature (43). This action occurs independently of granulosa cell proliferation induced by BMP2, highlighting the dual action capability of BMPs to inhibit and to induce cellular activity, and to induce differential responses during different stages of folliculogenesis.

*In vitro* culture of caprine granulosa cells with BMP2, 4, 6, and 7 reduces the percentage of DNA fragmentation (38). In the same study, FSHR silencing increased apoptosis. BMP4 neutralization in rodents causes an increase in apoptosis of the granulosa cells and the oocyte (37). BMP7 activated caspase 9 in granulosa cells; however, BMP4 and 7 had no effect on the levels of mitochondrial apoptotic factors, Bax and Bcl-x1 mRNA (25). Furthermore, BMP7 induced survivin and X-linked inhibitor of apoptosis protein to stimulate caspase 3 and 9 expression. BMP4, however, reduced factors downstream of caspase 3 that culminated in the reduction of apoptosis (Figure 3) (25).

## Regulation of BMPR1B and FSHR and Their Influence on Apoptosis

The BMP ligands are well established as regulators of granulosa cell proliferation in sheep (25, 44–48), cows (25, 49–52), and humans (3, 5, 22, 23, 53–56). In addition, granulosa cell apoptosis increases dramatically around the time of dominant follicle selection when the ratio of androgen is greater than estrogen (49, 57–59). The start of follicle selection coincides with the down-regulation of granulosa cell BMPR1B and FSHR expression (3, 4, 9).

In the transition from pre-antral to antral follicle, high levels of BMPs promote FSHR expression within the granulosa cells. The high levels of BMP6, 4, 7 (25), and BMP15 (60) concurrently sp suppress LHR expression in granulosa cells. During the subsequent follicle section process, the FSHR and BMPR1B expression is reduced along with the BMP ligands 4, 6, 7, and 15, and this may facilitate up-regulation of granulosa cell LHR expression (61). The follicles with granulosa cells expressing LHRs are able to continue to produce estrogens, and are recruited into the dominant cohort. FSHR is protective against apoptosis; therefore, as the FSHR level falls, apoptosis increases (4).

During continued growth of the selected dominant follicle, the estrogen levels rise to a critical level and trigger luteinization. Luteinization down-regulates the BMPR1B and the FSHRs in the ovulatory follicle. The reduction in FSHR and the cessation of proliferation momentarily reduces estrogen synthesis and the balance between proapoptotic and antiapoptotic factors. In older women, the dysregulation of granulosal FSHR and the BMPR1Bs would alter this balance and would be expected to change the apoptosis levels. An altered profile of BMPR1B expression in granulosa cells was also observed in young compared to older sheep (47, 62). Moreover, the BMPR1B mutation-induced reduction in apoptosis levels associated with the high ovulation rate in the Booroola sheep (47) was recently confirmed by the reduced GADD45A levels in the follicles of the Booroola sheep (62). (GADD45 alpha is a growth arrest and DNA-damage-inducible protein that is a gene marker for apoptosis.)

The cumulus granulosa cells surrounding the oocyte have reduced apoptosis levels compared to the mural granulosa cells (2, 63). The reduced apoptosis level is associated with the BMP ligand concentration gradient emanating from the oocyte (36). At the time of ovulation, luteinization expands the oocyte-cumulus complex, which closes the communication gap junctions between the oocyte and the cumulus cells. The disruption of the concentration gradient of BMPs results in an increase in the apoptosis level and reduced expression of LHR (64). Once luteinization has occurred, progesterone becomes the main antiapoptotic hormone.

## The Relationship Between Apoptosis and Oocyte Quality

In IVF programs it has long been recognized that fewer than 10% of oocytes collected become live births (65); hence, in the 1990s there was a concerted effort to find a marker for oocyte quality to better determine the chance of pregnancy. This led to investigations into the relationship between apoptosis and oocyte quality. Oocyte degeneration in the form of DNA fragmentation was linked to oocyte quality (66), and greater levels of DNA fragmentation were reported in the aged mouse oocyte. The level of granulosa cell apoptosis was increased in older IVF patients, similar to that observed in mice, and this was associated with a reduction in oocyte quality, fertilization, pregnancy, and live birth rate (1, 2, 67–69).

### Earlier Studies and Apoptosis Theory

The close proximity of the cumulus cells to the oocyte led to the investigation of cumulus cell apoptosis, and its relationship to fertility and oocyte quality. Lee et al. (70) found a strong association with fertility; however, conflicting results were also found (71). The level of cumulus cell apoptosis before the LH surge is reported to be less than 3% (2, 72). After the acquisition of LHR (73) and expansion of the cumulus away from the oocyte, apoptosis levels increase in both the oocyte and in the cumulus cells (71).

However, the techniques initially employed for evaluating apoptosis were based on light microscopy assessment of pyknotic cell count or index. What was clear from these early studies was the variance in development of the non-ovulatory smaller follicles compared to the larger preovulatory follicles (7). Nakahara et al. (2) went on to examine the effect of the subject’s age, and in contrast to other reports, they observed a reduced rate of granulosa cell apoptosis in the 40+ years age group. This finding was overshadowed by emphasis on the greater level of granulosa cell apoptosis in patients with a “poor ovarian responsiveness” to gonadotrophin stimulation. For example, when ovarian reserve was indirectly equated to the number of oocytes collected >12 mm in diameter, “independent of age,” the granulosa cell apoptosis rate was significantly greater. This implies that women with low ovarian reserve, or more correctly poor “ovarian responsiveness” to IVF stimulation, had greater levels of granulosa cell apoptosis.

Apoptosis levels within granulosa cells were also greater in the women who did not become pregnant, and was associated with patients with a high FSH level at the start of a cycle (implying poor ovarian reserve). Interestingly, when adjusted for the confounders of age and number of oocytes retrieved, granulosa cell apoptosis was related to pregnancy outcome and not the ovarian reserve (1). The results were significantly confounded by many uncontrolled variables and the methodology used to evaluate apoptosis had limitations.

### Recent Studies and Revised Apoptosis Theory

With the newer technique of flow cytometry, more granulosa cells could be examined (~5,000 per follicle) and the white blood cell fraction could be selectively removed using CD45 magnetic beads for subtraction gating to improve the accuracy of identifying granulosa cells. However, the common practice of pooling the granulosa cells collected from a range of follicle sizes from each patient precluded the identification of follicle size-related differences. Other errors were introduced by selectively discarding follicles because they were contaminated with blood and by the spectral overlap between fluorescein isothiocyanate and PI that was not compensated for during flow cytometry (1, 68).

In the current era of research, it is possible to individually analyze the granulosa cells collected from a single follicle of a known size determined using ultrasonography (5). The granulosa cell population can be selectively gated to remove contaminating white blood cells using a CD45 monoclonal antibody. Common leukocyte antigen; 7AAD (far red DNA stain) can be used in place of PI to minimize spectral overlap or flow cytometric compensation can be performed.

The recent study of Regan et al. (4, 5) examined the relationship between ovarian reserve, granulosa cell receptor density and apoptosis during healthy human follicle development. The effect of the age of women undergoing the IVF protocol on granulosa cell apoptosis rate related to cell surface gonadotropin receptor and BMPRIB receptor expression. The study showed that the apoptotic rate of granulosa cells was higher in follicles during the two critical stages of dominant follicle selection and the pre-ovulatory maturation stage of folliculogenesis in young women with better ovarian reserve, as determined by ultrasound-defined antral follicle counts (AFCs), compared to older women with a decreased AFC. The reduced apoptosis was associated with low levels of BMPR1B at the time of dominant follicle selection, whereas the lack of down-regulation of the BMPR1B, FSHR, and LHRs was associated with reduced granulosal apoptosis at the time of pre-ovulatory maturation (3, 4). The authors suggested that this result is a reflection of a poor mitogenic turnover rate of granulosa cells in healthy follicles in the older patients.

Notably, administration of gonadotropin-releasing hormone (GnRH) was shown to increase apoptosis of both cumulus and mural granulosa cells. However, in clinical practice, the predictability of the LH surge by its suppression with GnRH has outlived the importance of the finding (74); however, only Hoechst staining was performed for apoptosis determination in that study. This change may have been helped by the finding from another study that the apoptosis level in granulosa cells was not correlated with oocyte quality or fertilization rate, which was determined by using a new technique of identifying very early apoptosis *via* Annexin V and PI and flow cytometry (75). In another study using PI and Bcl2 fluorescent staining of granulosa cells in patients with the same ovarian reserve and age, the fertilization rate was the same but the patients (women) who achieved a successful pregnancy had a reduced apoptotic index (76). However, variation was very wide in the non-pregnant group (13.61 ± 9.26). Internalization of the phospholipid membrane (Annexin V assay) occurs after the caspase proteolytic cascade, but before DNA condensation and fragmentation (11). Unfortunately, unintentionally induced apoptosis by centrifuging cells at >300 *g* would limit the value of some studies (67, 74). In the study of Lee et al. (70), cumulus cell apoptosis was found to be greater in the 40+ years age group, corresponding to a poorer fertilization rate; however, only 200 cumulus cells were counted per cumulus-oocyte complex, and only four patients were in the older age groups.

As new techniques were developed, and their sensitivity increased, the granulosa caspase activity was also analyzed (71, 76–79). Yuan et al. (71) found no difference in TUNEL staining and caspase activity until the LH surge, whereas caspase activity for the combined caspases 1–9 was present before the LH surge but then disappeared after the LH surge (71). In a recent study, the same technique of counting pyknotic cells found that the level of apoptosis was reduced in patients who became pregnant (80). The finding was supported by qPCR data of a greater level of caspase 3 in the non-pregnant group. Although the results appear robust, an undisclosed number of follicles were sampled (between 1 and 4 per patient), and were not necessarily the corresponding follicle that produced the pregnancy. To confound the finding further, two embryos were transferred per patient; therefore, the outcome of the fate for each follicle, pregnant or not, was unclear.

At this time, the mechanisms of apoptosis were being investigated to determine whether various factors were proapoptotic or antiapoptotic (81). Whole ovary investigations also took place and revealed the level of DNA fragmentation (TUNEL assay) and caspase 3 activity to be undetectable in adult primordial, primary, and secondary follicles (59, 78, 82), whereas high levels of caspase-dependent apoptosis were recorded in antral follicles. However, in pre-ovulatory follicles, apoptosis was again rarely found (59). This change in apoptosis corresponds to the two critical times of FSHR down-regulation and LHR acquisition during dominant follicle selection; and later during pre-ovulatory down-regulation of LHR, FSHR, and BMPR1B (3, 5).

Early research by Sadraie et al. (67), reported that young patients produced more oocytes that were mature compared to the older age groups, which is consistent with the recently reported lack of down-regulation of BMPR1B, FSHR, and LHR, and final maturation of the follicle in older women (3–5). However, Jancar et al. (83), found no effect of granulosa cell apoptosis on fertilization rate or blastocyst development. Even though CD45-coated beads were used to purify the granulosa cells, they were pooled from multiple follicles. Moreover, the cells were centrifuged at 400 *g*, which is known to induce apoptosis. These factors may limit the significance of this study as well as a similar study examining granulosa cells from pooled follicles, where cells were also centrifuged at 400 *g* (84).

In older patients, the follicular levels of oxidative stress were increased, which can be associated with reduced oocyte quality (29). However, both the embryo and the oocyte produce reactive oxygen species (ROS) as a normal part of metabolism, and the production of the ROS may reflect other changes to the ovarian environment that are associated with aging and not necessarily a result of changed apoptosis levels (29, 70, 85–88).

Recently, in human fetal whole ovaries, granulosa cells from primordial follicles were reported to be positive for caspase 3 and DNA fragmentation (89). This limited semi-quantitative study also reported high levels of BAX in primordial follicles and Bcl2 in secondary follicles. However, the level of caspase dependent apoptosis was not commensurate with the expected loss of primordial follicles before birth or postnatally (89, 90). The inconsistent low levels of apoptosis indicate that other forms of caspase-independent cell death are in operation.

Several new proposals have surfaced in relation to the mechanisms of programed cell death that do not have features typically associated with apoptosis. These include autophagy (91), self-sacrifice to provide nutrients for neighboring follicles (92) and neuronal-endocrine induced apoptosis *via* the endoplasmic reticulum (93). Clearly, there are still many unanswered questions associated with the mechanism of cell death in the follicle.

## Conclusion

The complex control of folliculogenesis, ovulation, and the luteal phase of follicle development relies on the balance of proapoptotic and antiapoptotic factors to regulate cell survival. Apoptosis in the granulosa cell is predominantly *via* caspase-dependent signaling pathways, and the majority of apoptosis results in terminal differentiation of the granulosa cell at the antral surface of the follicle. The type of apoptosis and the signaling pathway depends on the stage of development of the follicle and the origin of the intrinsic or extrinsic trigger. A large volume of granulosa cell apoptosis within the granulosa membrana leads to death of the follicle.

Optimal receptor expression during folliculogenesis ensures maximum estrogen synthesis, which is essential for follicle survival. Cessation of estrogen driven-proliferation leads to ovulation, which imposes a new regulatory paradigm. The progesterone dominated luteal phase is also regulated by receptor expression to promote cell survival and corpora lutea function.

Using the techniques available at this time, it is apparent that apoptosis levels of the granulosa cells are reflective of the proliferative stage of the follicle rather than a predictor of oocyte health. Apoptosis is not used as a marker in the clinical setting because of its poor predictive capability regarding oocyte quality and ensuing pregnancy rate. Recent studies challenge the long-held view that increased granulosa cell apoptosis is typical of older patients and is related to oocyte quality. Rather, granulosa cell apoptosis appears to be an integral part of normal follicle development and reflects the mitogenic growth of the follicle that varies in a stage-dependant manner.

## Author Contributions

SR wrote the first draft of the manuscript and the final version of the article. PK, JY, YL, FA, and AD contributed to the draft of the manuscript, and critically revised the manuscript.

## Conflict of Interest Statement

The authors declare that the research was conducted in the absence of any commercial or financial relationships that could be construed as a potential conflict of interest.
